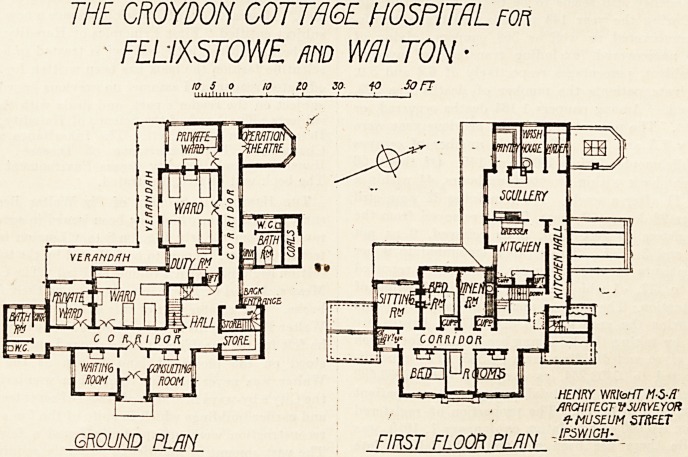# The Croydon Cottage Hospital for Felixstowe and Walton

**Published:** 1910-10-08

**Authors:** 


### THE CROYDON COTTAGE HOSPITAL FOR FELIXSTOWE AND WALTON.

In this excellently planned little hospital the whole of the accommodation for patients has been grouped together on the ground floor, and all the administrative part is on the floor above. By this arrangement the noise and the smell inseparable from the kitchen department has been kept well away from the patients.

The main entrance, which is on the south-east, has on one side a consulting-room, on the other a waiting-room. There are four wards, two for four beds, two for one bed, and these all face south or south-west, and all open into a verandah. A nurse's duty-room is placed centrally between the two large wards, and from here a lift goes up to the kitchen on the floor above.

A small operation theatre with a north light occupies the extreme north-west angle of the building. The only defect in the plan is the arrangement of the sanitary offices. There is, it is true, a cut-off lobby, but in it is placed the sink for emptying bed-pans—an arrangement which nullifies the effect of the lobby. Even in a small hospital such as this some more space is needed for dealing with bed-pans and other vessels used in the wards, and also for testing urine; and it is of course quite as essential to provide a proper cut-off to the sink room as to the w.c.

The upper floor is occupied by the kitchen offices, sitting room for nurses, and bedrooms for nurses and servants. Only one w.c. appears to be provided for both nurses and servants, and no bathroom. The building was designed by Mr. Henry Wright, M.S.A., architect, of Ipswich.

## Figures and Tables

**Figure f1:**